# Improving the efficiency of gene insertion in a human artificial chromosome vector and its transfer in human-induced pluripotent stem cells

**DOI:** 10.1093/biomethods/bpy013

**Published:** 2018-12-31

**Authors:** Yoshinori Hasegawa, Masashi Ikeno, Nobutaka Suzuki, Manabu Nakayama, Osamu Ohara

**Affiliations:** 1 Laboratory of Clinical Omics Research, Department of Applied Genomics, Kazusa DNA Research Institute, Chiba, Japan; 2 Aichi Medical University, Aichi, Japan; 3 Chromoresearch Inc., Aichi, Japan; 4 Laboratory of Medical Omics Research, Department of Frontier Research and Development, Kazusa DNA Research Institute, Chiba, Japan

**Keywords:** Human artificial chromosome (HAC) vector, hiPSC, dual RMCE

## Abstract

A human artificial chromosome (HAC) vector has potential to overcome the problems of stable gene expression associated with plasmid, transposon, and virus-based vectors, such as insertional mutagenesis, position effect, uncontrollable copy number, unstable gene expression, and DNA size limitation. The main advantages of the HAC are its episomal nature and ability to accommodate DNA inserts of any size. However, HAC vectors have two disadvantages: low efficiency of gene insertion and lack of reports regarding the successful HAC transfer to human-induced pluripotent stem cells (iPSCs). We here provide the first report of a method for the efficient transfer of HAC to human iPSCs for obtaining reproducible experimental results. Moreover, we achieved a 10% increase in the gene insertion efficiency in the HAC vector using our new site-specific recombination systems VCre/VloxP and SCre/SloxP.

## Introduction

Human-induced pluripotent stem cells (iPSCs) generated from the somatic cells of patients and donors provide new opportunities for research on human cell biology and disease modeling [1]. The ability of disease-specific iPSCs to differentiate into affected tissues can be used for *in vitro* disease modeling and development of cell therapeutics. Recent advancements in genome editing technology have considerably expanded the application of human iPSCs in disease modeling, drug testing, and screening, as well as in research on human cell biology [2, 3]. Knocking out genes in human iPSCs via genome editing, followed by inducible expression of transgenes such as variants of disease-associated genes are fairly effective strategies for understanding disease pathogenesis [4]. Virus and transposon-based vectors are most commonly used for the genetic manipulation of human iPSCs, although they are associated with several problems such as insertional mutagenesis, position effect, uncontrollable copy number, unstable gene expression, and DNA size limitation. Recently, the AAVS1 locus, a “safe-harbor” site with an open chromosomal region, has become more widely utilized as a transgene-targeting site in human iPSCs using transcription activator-like effector nuclease (TALEN) or clustered regularly interspaced short palindromic repeats (CRISPR)-Cas9, and reportedly showed stable transgene expression [5, 6]. Although the copy number of the transgene can be controlled and stable expression can be achieved, off-target cleavage is a potential drawback of these technologies.

As an alternative, human artificial chromosome (HAC)-based vectors are promising tools for overcoming the aforementioned problems associated with viral and non-viral vectors. HACs can be engineered using “top-down” [7] and “bottom-up” approaches [8, 9]. Using a bottom-up approach, we previously developed a HAC vector system in which one copy of the DNA fragment of interest can be manipulated by a Cre/lox insertion in any cell line of interest [10]. We used this system and the long interspersed nuclear elements 1 family of transposons to regulate chromatin [11], and developed a transgenic mouse harboring a HAC with a single copy of three human genes encoding cystathionine beta synthase, U2 auxiliary factor 1, and crystalline alpha A [12], respectively. We also established another transgenic mouse strain using this approach that retained multiple independent BAC transgenes [13].

Although several studies on HAC vectors have been reported [14, 15], two aspects need to be addressed for improving the features of HACs as gene expression vectors. First, to our knowledge, HAC transfer in human pluripotent stem cells such as human embryonic stem (hES) cells and hiPSCs has not been achieved to date, although one of the most suitable application areas of the HAC vector is gene therapy [16–18]. Second, although Cre recombinase-mediated cassette exchange (RMCE) utilizes the ability of site-specific recombinases to replace a HAC gene insertion site with a DNA fragment of interest, the gene insertion efficiency in the HAC vector is extremely low (10^−3^–10^−5^) [10, 13]. This efficiency is sufficient for generating an insertional clone of a gene of interest in the HAC vector using drug selection; however, construction of several clones for screening experiments using many plasmids at one Cre/lox recombination site is challenging. To solve the second problem, Anderson *et al*. [19] reported that the optimal configuration of *flp* and *cre* showed the highest level of dual RMCE (35–45% of plasmid-transfected cells). In contrast, our group developed two new site-specific recombination systems (VCre/VloxP and SCre/SloxP) for genome engineering [20]. These systems do not cross-react with the Cre/loxP and Flp/FRT systems, and therefore all four systems can be used together within the same cell. We attempted to use the VCre/VloxP and SCre/SloxP systems, along with the Cre/loxP and Flp/FRT systems, for dual RMCE. To address the first problem, an improved microcell-mediated chromosome transfer (MMCT) method of higher efficiency was recently reported [21]. MMCT enables a single intact mammalian chromosome or an autonomous megabase-sized chromosome fragment to be transferred from the donor to recipient cell lines. The maximum increase in MMCT efficiency obtained was approximately 6-fold when using colcemid and cytochalasin B with TN-16 + griseofulvin, and latrunculin B in combination with a collagen/laminin surface coating.

In this study, we aimed to solve these two major drawbacks limiting the utilization of HAC by using the VCre/VloxP and SCre/SloxP system and improving the MMCT technique. This approach provides a novel platform for an effective transgene-expression system in human iPSCs.

## Materials and methods

### Cell culture

Human epithelial kidney (HEK)293 and HEK293T cells were cultured in Dulbecco’s modified Eagle’s medium (DMEM) (Wako, Japan) supplemented with 10% fetal bovine serum (FBS) at 37°C and in an atmosphere of 5% CO_2_. Chinese hamster ovary (CHO) cells were maintained in Ham’s F-12 (Wako, Japan) with 10% FBS at 37°C in the presence of 5% CO_2_. The hiPSC line HPS0006, generated from skin tissue [22], was provided by the RIKEN BRC through the Project for Realization of Regenerative Medicine and the National Bio-Resource Project of the MEXT, Japan. The hiPSCs were maintained on iMatrix-511 (Nippi, Japan)-coated culture dishes in serum-free StemFit AK02N medium (Reprocell, Japan) at 37°C in the presence of 5% CO_2_.

### HAC vectors

**Figure 1:**
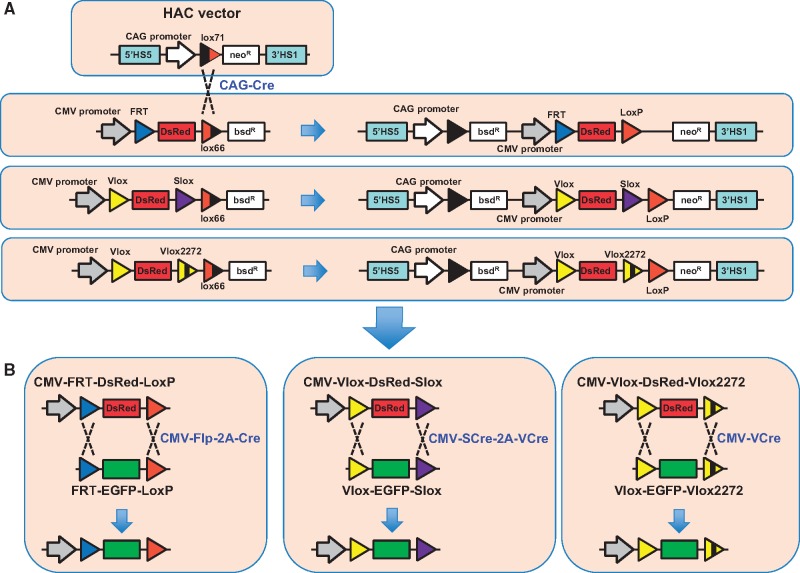
DsRed-expressing cassette insertion in the human artificial chromosome (HAC) and EGFP expression from the HAC using dual recombinase-mediated cassette exchange (RMCE). (**A**) Insertion of three DsRed-expressing cassettes in the HAC using Cre/lox recombination. (**B**) Exchange of DsRed with EGFP on the HAC using three types of dual RMCE.

The HAC vectors used in this study had multiple gene-expression cassettes, which were all constructed equally [10]: the expression cassettes consisted of a CAG promoter-driven neomycin resistance gene with mutant loxP, lox71, and human beta-globin 5’HS5 and 3’HS1 sites, which functioned as insulators [23] (Fig. 1A). We compared the dual RMCE efficiency among three DsRed-expressing cassettes: FRT-DsRed-loxP, Vlox-DsRed-Slox, and Vlox-DsRed-Vlox2272 (Fig. 1B). Vlox2272 is a mutant version of VloxP, and the two do not cross-react although VCre recombines at both sites, which enables simultaneous use of the VCre/VloxP and Cre/loxP systems within the same cell. Previously, Anderson *et al*. [19] compared the efficiency of dual RMCE using Flp and Cre among the following four configurations of transcription units: (i) *flp* and *cre* expressed as individual transcription units located on different vectors, (ii) *flp* and *cre* expressed as individual transcription units located on the same vector, (iii) *flp* and *cre* expressed from a single promoter and separated by an internal ribosome entry sequence, and (iv) *flp* and *cre* coding sequences separated by the 2A peptide and expressed as a single gene. They found that the Flp-2A-Cre plasmid driven by a single CMV promoter showed the highest efficiency. Considering these results, we constructed a CMV-SCre-2A-VCre plasmid for dual RMCE of the Slox-DsRed-Vlox cassette (Fig. 1B).

### Ethical approval

This study was approved by the Ethics Committee of the Kazusa DNA Research Institute.

### DsRed-expressing cassette insertion in the HAC vector

We constructed the DsRed-expressing cassettes, EGFP-cassettes, and recombinase plasmids by gene synthesis (Fig. 1A and B). For insertion of the DsRed-expressing cassettes into the HAC vector, 4 µg DsRed-expressing cassette was co-transfected with 1 µg CAGGS-Cre into 80%-confluent HEK293 or CHO cells containing the HAC vector in a 6-cm dish using FuGENE HD (Promega, Madison, WI) according to the manufacturer’s instructions. HEK293 or CHO cell lines were selected using 18 µg/ml or 5 µg/ml blasticidin S (Wako, Japan), respectively.

### Dual RMCE

Dual RMCE reactions were performed in accordance with the methods described by Anderson *et al*. [19]. Two micrograms of EGFP cassettes and 0.4 µg recombinase plasmids were co-transfected into the 80%-confluent cells on 6-well plates using Trans-IT 293 (Mirus, Madison, WI) according to the manufacturer’s instructions. The transfection efficiency of plasmids in HEK293 cells was estimated separately by transfecting the cells with a CMVpromoter-driven EGFP plasmid, and then the number of green fluorescent cells was counted. One-sixth of the cells co-transfected with the reporters and the expression vectors were transferred to a 6-well plate 24 h post-transfection, and the green fluorescent cells were counted 48 h post-transfection.

### Isolated metaphase chromosome transfection

**Figure 2:**
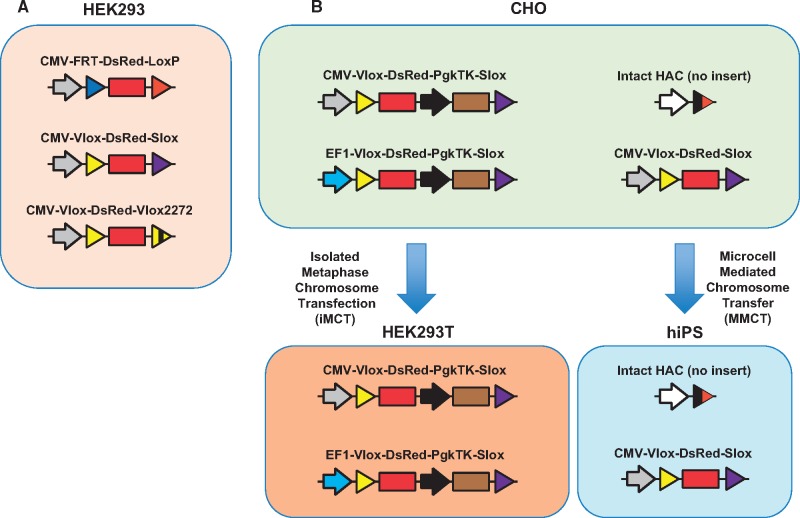
Cell lines containing HACs generated in this study, and HAC transfer. (**A**) Three HEK293 cell lines containing HACs with DsRed-expressing cassettes. (**B**) Four CHO cell lines containing HACs and HAC transfer to HEK293T cell lines by iMCT or to hiPSCs by MMCT.

Isolated metaphase chromosome transfection (iMCT) from CHO cells to HEK293T cells (Fig. 2B) was performed as described previously [24]. In brief, 70%-confluent CHO cells cultured on eight 10-cm dishes were arrested in metaphase using 0.1 µg/ml colcemid in culture medium. The mitotic cells were then swelled in 75 mM KCl for 10 min at room temperature. The cells were then washed in polyamine (PA) buffer (15 mM Tris–HCl, pH 7.4, 0.2 mM spermine, 0.5 mM spermidine, 2 mM EDTA, 0.5 mM EGTA, 80 mM KCl, 20 mM NaCl) and resuspended in cold PA buffer containing 0.1% digitonin. The swollen cells were passed through a 10-ml syringe with a 27-gauge needle to release the chromosomes. The 80%-confluent HEK293T cells growing on one 6-cm dish were transfected using FuGENE HD according to the manufacturer’s instructions. Stable transfectants were screened using 18 µg/ml blasticidin S.

### MMCT

CHO cells were grown to 70% confluence on twenty 10-cm dishes, and then cultured for 72 h in Ham’s F-12 medium containing colcemid (0.05 µg/ml). The collected cells were incubated in serum-free DMEM containing cytochalasin B (20 µg/ml) for 5 min at 37°C, followed by addition of an equal volume of Percoll (Amersham Biosciences, CA, USA). The suspension was centrifuged in a Hitachi R20A2 rotor at 15 000 rpm for 90 min at 37°C. The collected microcells containing the HAC were mixed with hiPSCs from four 10-cm dishes and centrifuged at 2000 rpm for 5 min. The pellet was suspended in 1 ml 50% PEG1500 (Roche) and incubated at room temperature for 90 s. The fused cells were washed twice and plated onto iMatrix-511-coated 10-cm dishes. hiPSCs containing the HAC were selected using 40 µg/ml G418 (Roche). The key difference between our usual MMCT and our improved MMCT is the use of TN-16 (160 µM, Santa Cruz, CA, USA) and griseofulvin (50 µM, Santa Cruz, CA, USA) instead of colcemid for cell synchronization at metaphase [21].

### Fluorescent *in situ* hybridization

Metaphase chromosome spreads were prepared on glass slides after methanol/acetate (3:1) fixation. Fluorescent *in situ* hybridization (FISH) analyses were performed using pBelo-BAC DNA and 11-4 alphoid DNA as probes for detecting HAC vectors [25] according to conventional methods. Biotin-labeled 11-4 alphoid DNA was visualized using FITC-conjugated avidin (Vector Laboratories), and digoxigenin-labeled pBelo-BAC DNA was visualized using TRITC-conjugated anti-digoxigenin antibody (Roche).

### Total RNA extraction, mRNA library preparation, and 3′ RNA-seq

It is imperative to maintain hiPSCs in an undifferentiated state without any pathogen contamination for therapeutic applications. Therefore, we compared the whole-transcriptome profiles between control iPSCs and iPSC transformants containing HACs, with focus on the expression of pluripotent markers using 3′ RNA-seq. Total RNA was extracted from 50%-confluent cells on a 3.5-cm dish and each sample was transferred to 800 µl TRIZOL reagent (Life Technologies, Carlsbad, CA, USA). The solution was vigorously vortexed and incubated at room temperature for 5 min. The solution was then centrifuged after adding 200 µl chloroform, and the aqueous phase was carefully transferred to a new tube, following which 10 µg glycogen (Life Technologies) was added as a co-precipitant. RNA was precipitated by adding 600 µl isopropyl alcohol. The RNA pellet was washed once with 75% ethanol and then dissolved in 10 µl RNase-free water. The concentration and the quality of the RNA were verified using a Qubit fluorometer (Life Technologies) and Agilent 2100 bioanalyzer, respectively. Purified total RNA (500 ng) was used for RNA library preparation, according to the instructions of the Quant Seq 3′ mRNA-seq library preparation kit FWD of Illumina (Lexogen, Vienna, Austria). The RNA libraries were sequenced on an Illumina NextSeq 500 system with 75-nt-long reads. The raw data were deposited in the DNA Data Bank of Japan (DDBJ; accession nos. DRA007133, PRJDB7263).

### 3′ RNA-seq data analysis

Prior to assembly, the adapter sequences were removed from the raw reads, and the trimmed reads were assessed for quality using the FASTX toolkit (v0.0.13) software. Base trimming was performed from the 3′ end of each read to remove bases with quality below Q30 up to a minimum length of 25 bp. Reads shorter than 25 bp were removed prior to further analysis. Each read was mapped to the human genome hg38 using Strand NGS (v3.1, Agilent Technologies) and default settings. After normalization of DEseq [26] using default settings, scatter plots were generated based on gene expression patterns.

### Mitotic stability

The mitotic stability of the HAC in hiPSCs was evaluated by culturing the cells for two weeks under non-selective growth conditions. After 2-week culture in the absence of G418, spreads of metaphase chromosomes were prepared, and the presence of HACs was analyzed by FISH as described above. Specifically, the percentage of metaphase cells with HAC among a total of 50 spreads was calculated. The daily rate of HAC loss (*R*) was then calculated by the following formula: *N*_14_ = *N*_0_ × (1 − *R*)^14^, where *N*_0_ is the number of metaphase spreads containing a HAC under selective conditions, and *N*_14_ is the number of metaphase spreads containing a HAC after 2 weeks of culture under non-selective conditions.

## Results

### Efficiency of dual RMCE

**Figure 3:**
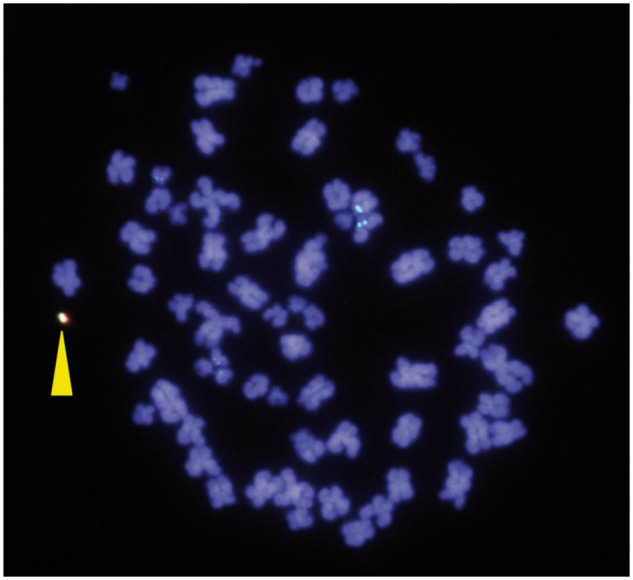
FISH analysis of HEK293 cells after gene loading on the human artificial chromosome (HAC) by dual recombinase-mediated cassette exchange (RMCE). The HAC is labeled green (alphoid probe) and red (BAC probe), and indicated by the yellow triangle.

After successful insertion of the DsRed-expressing cassettes in the HAC vector by Cre-lox recombination of the lox66 site at the DsRed-expressing cassettes and the lox71 site in the gene expression cassette (Fig. 1A), we confirmed that the vector was maintained extrachromosomally in the host cell using FISH (Fig. 3).

**Figure 4:**
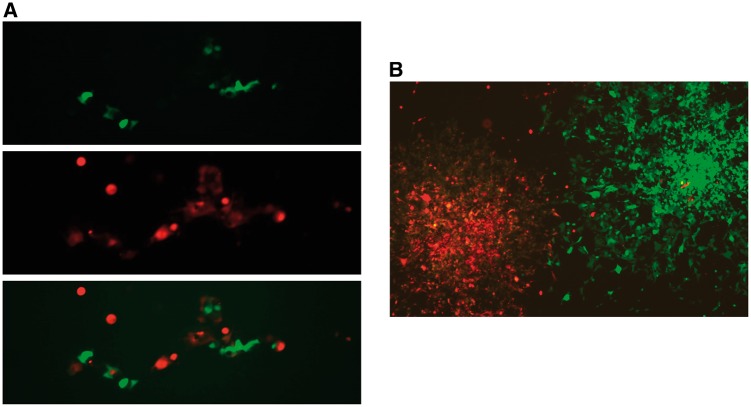
HEK293 cells after dual recombinase-mediated cassette exchange (RMCE). (**A**) Two days after dual RMCE. (**B**) Three weeks after dual RMCE. Two colonies originated from two cells: one was intact (DsRed) and the other was cassette-exchanged (EGFP).

**Figure 5:**
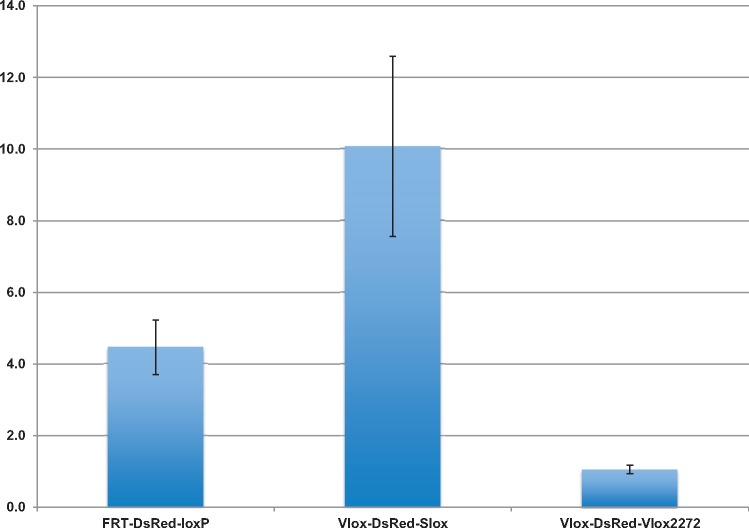
Efficiency of dual recombinase-mediated cassette exchange. The blue bars show the mean value of three experiments; the error bars indicate SD.

The dual RMCE activities of the three cassettes, FRT-DsRed-LoxP, Vlox-DsRed-Slox, and Vlox-DsRed-Vlox2272, were analyzed by assessing the efficiency of the replacement of a DsRed-expressing cassette, which was pre-integrated into the HAC vectors with a promoterless EGFP cassette located on an incoming vector (Fig. 1B). We did not observe the green fluorescent cells (expressing EGFP) when only the EGFP cassette was transfected. In contrast, after the EGFP cassettes and the recombinase-encoding plasmids were co-transfected, green fluorescent cells could be detected and the cells were negative for red fluorescence (Fig. 4A). Among the three cassettes tested, the Vlox-DsRed-Slox cassette showed the highest efficiency (10%) (Fig. 5). The efficiency was twice and ten times as that of the FRT-DsRed-loxP and Vlox-DsRed-Vlox2272 cassettes, respectively, and the corrected efficiency observed in the plasmid-transfected cells reached ∼20%, as the estimated transfection efficiency of CMV-promoter-driven EGFP plasmid in HEK293 cells was 50–52% in each experiment. EGFP was stably expressed 3 weeks after EGFP was inserted in the HAC by dual RMCE (Fig. 4B).

### HAC transfer

**Figure 6:**
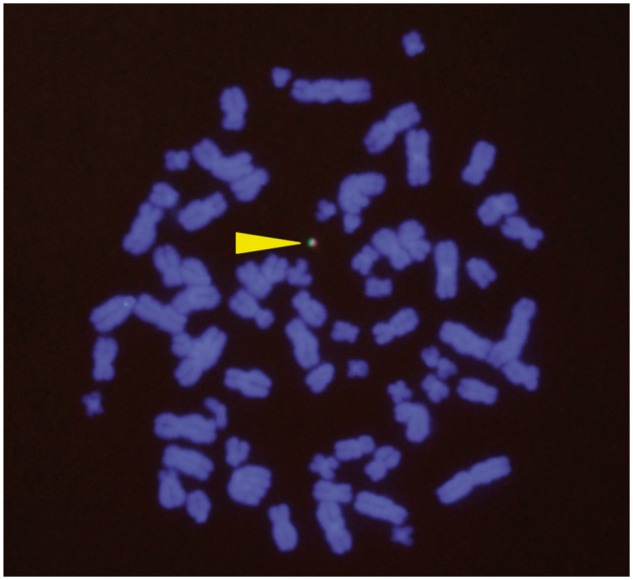
FISH analysis of HEK293T cells after isolated mitotic chromosome transfection of a human artificial chromosome. FISH analysis was performed using the alphoid probe (green) and BAC (red) (see details in the section “Materials and Methods”). The HAC is indicated by the yellow triangle.

**Figure 7:**
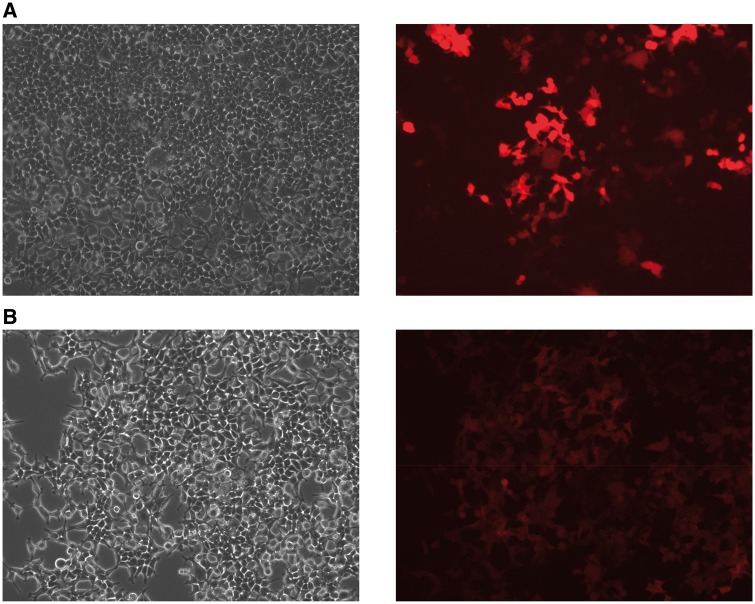
DsRed expression levels from the HAC in HEK293T cells. (**A**) CMV promoter-driven expression of DsRed. (**B**) EF1 promoter-driven expression of DsRed.

**Figure 8:**
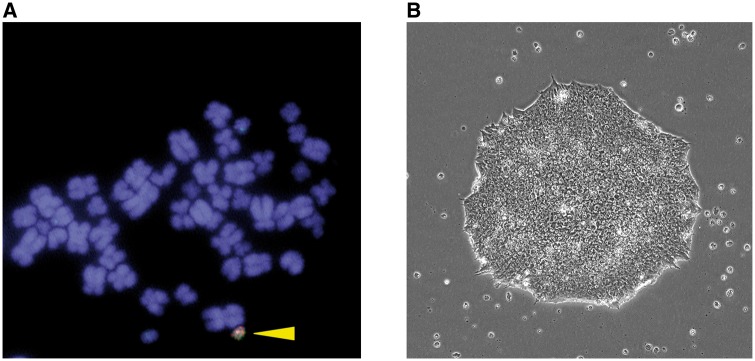
Human iPSCs after microcell-mediated chromosome transfer of human artificial chromosome. (**A**) FISH analysis. The HAC is labeled green (alphoid probe) and red (BAC probe) and is indicated by a yellow triangle. An arrowhead shows the HAC. (**B**) Phase-contrast image.

As the characteristics of cultured cells, including PSCs and established cell lines, change during prolonged culture, maintenance of cells in short passages is recommended. In fact, all transgenic mice harboring additive genes on the HAC were generated from mouse ES cells, which transferred the HAC with genes from donor cells such as CHO cells [12, 13]. Our transgenic mouse retaining multiple independent BAC transgenes possessed two consecutive gene insertions on the HAC in CHO cells following MMCT transfer into mouse ES cells [13]. We routinely conduct iMCT for HAC transfer because it is sufficient only for one 6-cm dish of 70–80%-confluent recipient cells and eight 10-cm dishes of 70–80%-confluent donor cells containing HACs. However, the transfection efficiency of plasmids in hiPSCs using transfection reagents is poor; the transfection efficiency was <5% when a CMV-promoter driven EGFP plasmid was transfected in the hiPSC line HPS0076 using FuGENE HD. Therefore, we used iMCT for HAC transfer from CHO to HEK293T cells, and we used MMCT for transfer from CHO to hiPSCs (Fig. 2B). We obtained over 20 clones per trial of iMCT for HEK293T in two trials (Fig. 6), which showed promoter-dependent DsRed expression as described previously [10] (Fig. 7). By contrast, only four clones were obtained for HAC/CMV-Vlox-DsRed-Slox (Fig. 8) and no clone for intact HAC was obtained using MMCT in two trials with hiPSCs. These results showed that our conventional MMCT method was not suitable for the stable transfection of hiPSCs, and that it is not applicable for general HAC transfer to hiPSCs. Therefore, we developed an improved MMCT for hiPSCs and successfully obtained over 20 hiPSCs harboring intact HAC in a single trial.

### Expression of control iPSCs and iPSC transformants

**Figure 9:**
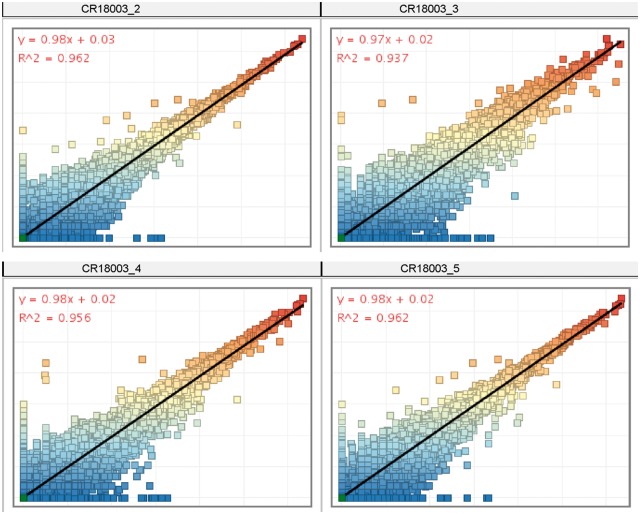
3′ RNA-seq expression scatterplots between two representative samples of control hiPSCs and hiPSCs containing HACs. The plot is on a log2-transformed scale.

We conducted feeder-free and xeno-free culture for hiPSC expansion, and confirmed the maintenance of pluripotency during culture in the control cells and transformants using 3′ RNA-seq for pluripotent marker genes. The RIN value of extracted total RNA of all samples was ≥9. 3′ RNA-sequencing generated 11,115,860 raw reads from five RNA samples using the Illumina NextSeq platform, with approximately 2.2 million reads per sample (Table 1). After discarding adapters and low-quality reads from the raw data, 7,562,155 clean reads (68.0%) were obtained, 90.1–91.6% of which were mapped to the hg38 human genome. Scatter plots of the normalized 3′ RNA-seq expression data showed high correlation between control iPSCs and all four iPSC transformants (*R*^2^ > 0.93) (Fig. 9). Furthermore, the pluripotent stem cell markers *OCT3/4, SOX2, NANOG*, and *SSEA1* were expressed in control iPSCs and four iPSC transformants (Supplementary Table S1), whereas the HAC-selectable gene encoding neomycin resistance was expressed in all iPSC transformants but not in control iPSCs (data not shown).

**Table 1:** Summary of 3′ RNA-seq data analysis.

Sample ID	Sample name	Raw reads	Clean reads	Mapped reads
CR18003-1	Control hiPSC (HPS0076)	2,255,004	1,595,266	1,453,559
CR18003-2	hiPSC/HAC #1	2,309,650	1,518,786	1,371,336
CR18003-3	hiPSC/HAC #2	2,095,731	1,456,552	1,312,358
CR18003-4	hiPSC/HAC #3	2,065,626	1,468,999	1,326,291
CR18003-5	hiPSC/HAC #4	2,389,849	1,522,552	1,394,054

### Mitotic stability of the HACs

We examined the mitotic stability of HAC in the four clones used in the 3ʹ RNA-seq experiments using FISH. After 2 weeks without selection, 100%, 98%, 96%, and 100% of the metaphase cells in hiPSC/HAC#1, hiPSC/HAC#2, hiPSC/HAC#3, and hiPSC/HAC#4 retained the HAC, respectively, and the number of HAC copies per cell was maintained at one under non-selective conditions. The daily rate of HAC loss was 0%, 0.14%, 0.29%, and 0% for hiPSC/HAC#1, hiPSC/HAC#2, hiPSC/HAC#3, and hiPSC/HAC#4, respectively. No integration of the HAC into host chromosomes was observed in any cell line. These results indicated that the HAC can be maintained stably in hiPSCs.

## Discussion

We successfully transferred a HAC from donor cells into hiPSCs using MMCT. This represents the first report of HAC transfer into hPSCs. To date, two methods for generating HAC-containing hPSCs have been reported. The first method developed by Oshimura’s group involved constructing a HAC with a complete 2.4-Mb genomic dystrophin sequence (DYS-HAC) followed by transformation in DT40 and CHO cells [27]. The DYS-HAC was transferred to Duchenne muscular dystrophy patient-derived fibroblasts from CHO cells, from which iPSCs were generated using a combination of lentiviral infection for expressing mouse SLC7A1 and retroviral infection for expressing KLF4, SOX2, OCT4, and c-MYC [28]. The other method involved *de novo* HAC generation in ES cells using HSV-1 amplicon technology [29]. In contrast, we succeeded in directly transferring the HAC with limited genes to hiPSCs. The proposed transfer method can become a superior approach for the research and application of established pluripotent cell lines, such as in drug discovery, toxicity testing, disease modeling, and gene therapy. Furthermore, the efficiency of the HAC transfer in hiPSCs via MMCT was sufficiently increased to be applicable to any iPSC line; indeed, we have already successfully achieved HAC transfer with two other hiPSC lines (unpublished data). Importantly, strong correlations between gene expression levels were observed between control iPSCs and the iPSC transformants.

There are significant differences between our MMCT method and that used by Liskovykh *et al*. [21]. The first difference involves the microcell collection after micronucleation. In our method, the microcell was centrifuged in cell culture medium mixed with Percoll, whereas Liskovykh and colleagues performed this step in only the cell culture medium. The second difference lies in the reagent used for the fusion of microcells and recipient cells. We used PEG1500, whereas Liskovykh and colleagues used the HVJ envelope cell fusion kit (Cosmo Bio Ltd., Japan). However, since the efficiency of their basal MMCT was similar to that of our usual MMCT method, we changed only one common reagent for the improved method to induce micronucleation: colcemid to TN-16 and griseofulvin. This change significantly increased the efficiency of HAC transfer to recipient cells in three different human cell lines by ∼3.7-fold [21]. Moreover, the advantages of iMCT include the use of small amounts of recipient and donor cells and lack of host cell limitation. We previously reported the successful transfer of a HAC vector containing the human beta globin gene cluster from K562 cells to HT1080 or HeLa cells by iMCT, and the expression of the Gγ- and Aγ-globin genes from the HAC was activated in transformed K562 cells and was reproducibly repressed in non-erythrocyte cell lines (HeLa and K562) [24]. It is likely that we selected the appropriate donor cells for HAC vector transformation; in other words, iMCT or MMCT should be used for HAC vector transfer in hiPSCs depending on the purpose. Currently, certain commercial transfection reagents with high efficiency of plasmid transfection in hiPSC and hES cells are available. We intend to use these reagents for HAC vector transfer in hiPSCs via iMCT. Nevertheless, we are in the process of testing the application of a HAC vector for gene therapy, especially for genetic disorders, using disease-specific hiPSCs.

Dual RMCE of VCre/VloxP and SCre/SloxP systems with the VloxP-DsRed-SloxP cassette (VDS) showed the highest efficiency (20% of the plasmid-transfected cells) in this study. This efficiency was approximately half that obtained by Anderson *et al*. [19], who observed that the FRT-DsRed-LoxP cassette (FDL) yielded the maximum transformation efficiency. They further reported that the efficiency of FDL was twice as high as that of the LoxP-DsRed-FRT cassette (LDF). As generation of FDL in a HAC vector is difficult because of the construction of the HAC gene insertion cassette (Fig. 1A), we tested LDF, VDS, and the VLoxP-DsRed-VLoxP2272 cassette in HAC in this study. The efficiency of VDS was twice as high as that of LDF under similar conditions, and the dual RMCE efficiency of VDS was almost equal to that of FDL. One of the possible reasons for the differences between the two results may be related to the different characteristics of the cells used between studies, that is, CHO cells versus HEK293 cells (this study). Nevertheless, this high transfection efficiency enabled us to perform a screening experiment for many cells expressing single copies of the genes of interest in one cell per trial of transfection; in addition, stable transformants could be generated in the absence of drug selection. We are planning to conduct functional screening analyses with many mutants of disease-associated genes to express the genes in human disease-specific iPSCs using HAC-containing VDS cassettes. Furthermore, we are developing a screening system for genetic interaction using abundant KAZUSA cDNA and Halo-tag clones, as the combination of HAC technology and Halo-tag clones was reported to be successful for centromere chromatin assembly [30]. Moreover, the Vlox-Slox cassette system can be applied for integration in the host genome, and we have already generated HEK293 cells containing one CMV-Vlox-DsRed-Slox cassette at the AAVS1 safe harbor site using specific TALEN-mediated homologous recombination.

In conclusion, we believe that HAC vectors will offer an effective transgene expression system in human iPSCs.

## Supplementary Material

Supplementary Table S1
